# The Light Skin Allele of *SLC24A5* in South Asians and Europeans Shares Identity by Descent

**DOI:** 10.1371/journal.pgen.1003912

**Published:** 2013-11-07

**Authors:** Chandana Basu Mallick, Florin Mircea Iliescu, Märt Möls, Sarah Hill, Rakesh Tamang, Gyaneshwer Chaubey, Rie Goto, Simon Y. W. Ho, Irene Gallego Romero, Federica Crivellaro, Georgi Hudjashov, Niraj Rai, Mait Metspalu, C. G. Nicholas Mascie-Taylor, Ramasamy Pitchappan, Lalji Singh, Marta Mirazon-Lahr, Kumarasamy Thangaraj, Richard Villems, Toomas Kivisild

**Affiliations:** 1 Department of Evolutionary Biology, Institute of Molecular and Cell Biology, University of Tartu, Tartu, Estonia; 2 Estonian Biocentre, Tartu, Estonia; 3 Division of Biological Anthropology, University of Cambridge, Cambridge, United Kingdom; 4 Institute of Mathematical Statistics, University of Tartu, Tartu, Estonia; 5 CSIR-Centre for Cellular and Molecular Biology, Hyderabad, India; 6 School of Biological Sciences, University of Sydney, Sydney, Australia; 7 Leverhulme Centre for Human Evolutionary Studies, University of Cambridge, Cambridge, United Kingdom; 8 Chettinad Academy of Research and Education, Chettinad Health City, Chennai, India; 9 Banaras Hindu University, Varanasi, India; 10 Estonian Academy of Sciences, Tallinn, Estonia; University of Washington, United States of America

## Abstract

Skin pigmentation is one of the most variable phenotypic traits in humans. A non-synonymous substitution (rs1426654) in the third exon of *SLC24A5* accounts for lighter skin in Europeans but not in East Asians. A previous genome-wide association study carried out in a heterogeneous sample of UK immigrants of South Asian descent suggested that this gene also contributes significantly to skin pigmentation variation among South Asians. In the present study, we have quantitatively assessed skin pigmentation for a largely homogeneous cohort of 1228 individuals from the Southern region of the Indian subcontinent. Our data confirm significant association of rs1426654 SNP with skin pigmentation, explaining about 27% of total phenotypic variation in the cohort studied. Our extensive survey of the polymorphism in 1573 individuals from 54 ethnic populations across the Indian subcontinent reveals wide presence of the derived-A allele, although the frequencies vary substantially among populations. We also show that the geospatial pattern of this allele is complex, but most importantly, reflects strong influence of language, geography and demographic history of the populations. Sequencing 11.74 kb of *SLC24A5* in 95 individuals worldwide reveals that the rs1426654-A alleles in South Asian and West Eurasian populations are monophyletic and occur on the background of a common haplotype that is characterized by low genetic diversity. We date the coalescence of the light skin associated allele at 22–28 KYA. Both our sequence and genome-wide genotype data confirm that this gene has been a target for positive selection among Europeans. However, the latter also shows additional evidence of selection in populations of the Middle East, Central Asia, Pakistan and North India but not in South India.

## Author Summary

Human skin color is one of the most visible aspects of human diversity. The genetic basis of pigmentation in Europeans has been understood to some extent, but our knowledge about South Asians has been restricted to a handful of studies. It has been suggested that a single nucleotide difference in *SLC24A5* accounts for 25–38% European-African pigmentation differences and correlates with lighter skin. This genetic variant has also been associated with skin color variation among South Asians living in the UK. Here, we report a study based on a homogenous cohort of South India. Our results confirm that *SLC24A5* plays a key role in pigmentation diversity of South Asians. Country-wide screening of the variant reveals that the light skin associated allele is widespread in the Indian subcontinent and its complex patterning is shaped by a combination of processes involving selection and demographic history of the populations. By studying the variation of *SLC24A5* sequences among a diverse set of individuals, we show that the light skin associated allele in South Asians is identical by descent to that found in Europeans. Our study also provides new insights into positive selection acting on the gene and the evolutionary history of light skin in humans.

## Introduction

Human skin color varies widely among and within populations and is a classic example of adaptive evolution. Skin pigmentation in humans is largely determined by the quantity and distribution of the pigment melanin, which is packed in melanosomes and then transferred from melanocytes (melanin-forming cells) to the surrounding epidermal keratinocytes [1]. Human melanin is primarily composed of two distinct polymers: eumelanin (brown/black) and pheomelanin (yellow/red), which differ in their physical properties and chemical composition [2]. In addition to the amount and type of melanin, other factors such as the size, shape, number, and cellular distribution of melanosomes also contribute to the variation in skin color [3]. Comparative studies of model organisms, pigmentation disorders and genome-wide studies have played a key role in the identification of human pigmentation genes [4]–[7]. A total of 378 candidate loci, including 171 cloned genes, are currently recorded in the Color Genes database (http://www.espcr.org/micemut/), yet only a few of them have been confirmed to have potentially function-altering polymorphisms in humans.

A significant correlation between skin color and ultraviolet radiation (UVR) levels observed at the global scale suggests that natural selection plays an important role in determining the distribution of this phenotypic trait [8]. The evolution of dark skin at low latitudes has been mainly accredited to the requirement of photo-protection against UVR which causes sunburn and skin cancer, whereas the evolution of light skin has been most commonly associated with vitamin D deficiency [9], [10]. It has been proposed that as humans started to colonize higher latitudes, where UVR levels were lower, dark skin could not absorb sufficient UVR for efficient vitamin D synthesis, hence natural selection favored the evolution of light skin [8], [11]. This is indirectly supported by the observation that candidate pigmentation genes are collectively enriched by high-F_ST_ single-nucleotide polymorphisms (SNP) [12]–[14]. Furthermore, data mining of publicly available datasets, such as HapMap, Perlegen and Human Genome Diversity Project (HGDP), has provided evidence of selection signals in pigmentation-related genes in one or more populations (see [15] and references therein), [16] thus elucidating the history of human adaptation to local environments for this complex trait.

One of the key pigmentation genes in humans is *SLC24A5* (OMIM 609802). It is located on chromosome 15q21.1 and encodes a protein called NCKX5. The association of this gene with lighter pigmentation was initially discovered in zebrafish [4]. Using admixed populations, it was further demonstrated in this study [4] that a non-synonymous variant (ref SNP ID: rs1426654) in the third exon of this gene explains 25–38% of the skin color variation between Europeans and West Africans. The ancestral (G) allele of the SNP predominates in African and East Asian populations (93–100%), whereas the derived (A) allele is almost fixed in Europe (98.7–100%) [4]. Functional assays of this gene suggested its direct involvement in human melanogenesis through cation-exchange activity [17], [18]. However, the fact that the ancestral (G) allele is virtually fixed not only in Africans but also in East Asians suggests that light skin at high latitudes evolved independently in East and West Eurasia [19]. Genome-wide scans have also identified *SLC24A5* as one of the most important “hot spots” for positive selection in Europeans, thereby supporting the role of natural selection acting on this gene [4], [20], [21].

Populations of South Asia live at lower latitudes than would be expected to require selection for lighter skin color on the basis of improved vitamin D synthesis [8]. Nevertheless, South Asians do exhibit a wide variation in skin color [22]. Two previous studies have assessed the genetics of skin pigmentation variation in expatriates from South Asia. The first of these [6] concluded that non-synonymous variants at three genes, *SLC24A5*, *SLC45A2* (OMIM 606202), and *TYR* (OMIM 606933), collectively contribute to variation in skin pigmentation in South Asians, with *SLC24A5* showing the largest effect. The second study on common disease variants suggested high prevalence of the light skin associated allele of *SLC24A5* in Asian Indians [23]. Nevertheless, both the studies involved populations that were structured and represented only a small range of the vast ethnic and genetic landscape of South Asia. Hence, comprehensive assessment of this phenotypic trait in native populations of South Asia has been lacking so far.

Therefore, in the present study, we sought to address the following objectives. First, we aimed to quantify the amount of skin pigmentation variation that can be explained by the rs1426654 SNP of *SLC24A5* in a homogeneous cohort of 1228 individuals from South Asia. Second, we studied the geospatial pattern of rs1426654-A allele in the Indian subcontinent using 1573 individuals from 54 populations and investigated how various factors influence its distribution. Third, we aimed to uncover the fine-scale genetic variation of *SLC24A5* and determined the coalescence age of rs1426654 by resequencing 11.74 kb in a diverse set of 95 individuals. Lastly, we assessed whether *SLC24A5* resequencing data and genome-wide genotype data were in concordance with the earlier reported evidence of positive selection in Europeans, and tested for any further evidence of selection among the studied populations. Our results confirm that rs1426654 plays a key role in pigmentation variation, while in-depth study of the light skin associated allele (rs1426654-A) among Indian populations reveals that the genetic architecture of skin pigmentation in South Asia is quite complex. The present study also provides important insights on evidence of positive selection and the evolutionary history of this light skin associated allele.

## Results

### Variation of melanin index in South Asia and its association with rs1426654 SNP

Phenotypic assessment of melanin index (MI) across 1674 individuals from two distinct cohorts, Cohort A and Cohort B (see Materials and Methods; Tables 1, S1 and S2) demonstrated a wide variation in skin color (MI 28–79) in South Asia. Comparison with published datasets for the regions of the world revealed that the observed range in South Asians was three times greater than that in East Asians and Europeans and comparable to that of Southeast Asians (Table 1). Notably, Cohort A (n = 1228) which included individuals from three closely related agricultural castes of Andhra Pradesh in South India, shows remarkable variation in skin color (MI 30–64), similar to heterogeneous pool of samples in Cohort B (MI 28–79).

**Table 1:** Global range of human skin pigmentation assessed by the melanin index (MI).

Population	Sampling location	No of individuals	MI Average	MI Range (Min-Max)	Reference
South Asia, Cohort A	India	1228	43.6	30–64	this study
Kapu	Andhra Pradesh	272	43.4		this study
Naidu	Andhra Pradesh	112	43.6		this study
Reddy	Andhra Pradesh	844	43.7		this study
South Asia, Cohort B	India	446	45.7	28–79	this study
Kurumba	Tamil Nadu	39	56.1		this study
Badaga	Tamil Nadu	47	44.7		this study
Korku	Maharashtra	64	53.2		this study
Kota	Tamil Nadu	46	44.6		this study
Nihali	Maharashtra	63	56.9		this study
Ror	Haryana	56	41.7		this study
Toda	Tamil Nadu	43	43.3		this study
Brahmin	Tamil Nadu	22	41.4		this study
Saurashtrian	Tamil Nadu	36	41.9		this study
Yadava	Tamil Nadu	30	57.9		this study
African American	USA	232	53.4	32–80	Parra et al. 2004 [69]
African Caribbean	UK	173	57.8	38–80	Parra et al. 2004 [69]
Bougainville Island	Papua New Guinea	153	89.8	70–115	Norton et al. 2006 [45]
Orang Asli	Peninsular Malaysia	517	47.6	28–75	Ang et al. 2012 [33]
Negrito	Peninsular Malaysia	55	55.1	34–70	Ang et al. 2012 [33]
Senoi	Peninsular Malaysia	412	45.5	28–75	Ang et al. 2012 [33]
Proto Malay	Peninsular Malaysia	50	42.2	30–61	Ang et al. 2012 [33]
East Asian	USA	9	31.8	28–36	Shriver et al. 2000 [70]
European	Europe	469	29	20–39	Candille et al. 2012 [5]
Mexican	Mexico	156	46.1	36–56	Parra et al. 2004 [69]
Puerto Rican	USA	64	36.8	26–55	Parra et al. 2004 [69]

**Figure 1:**
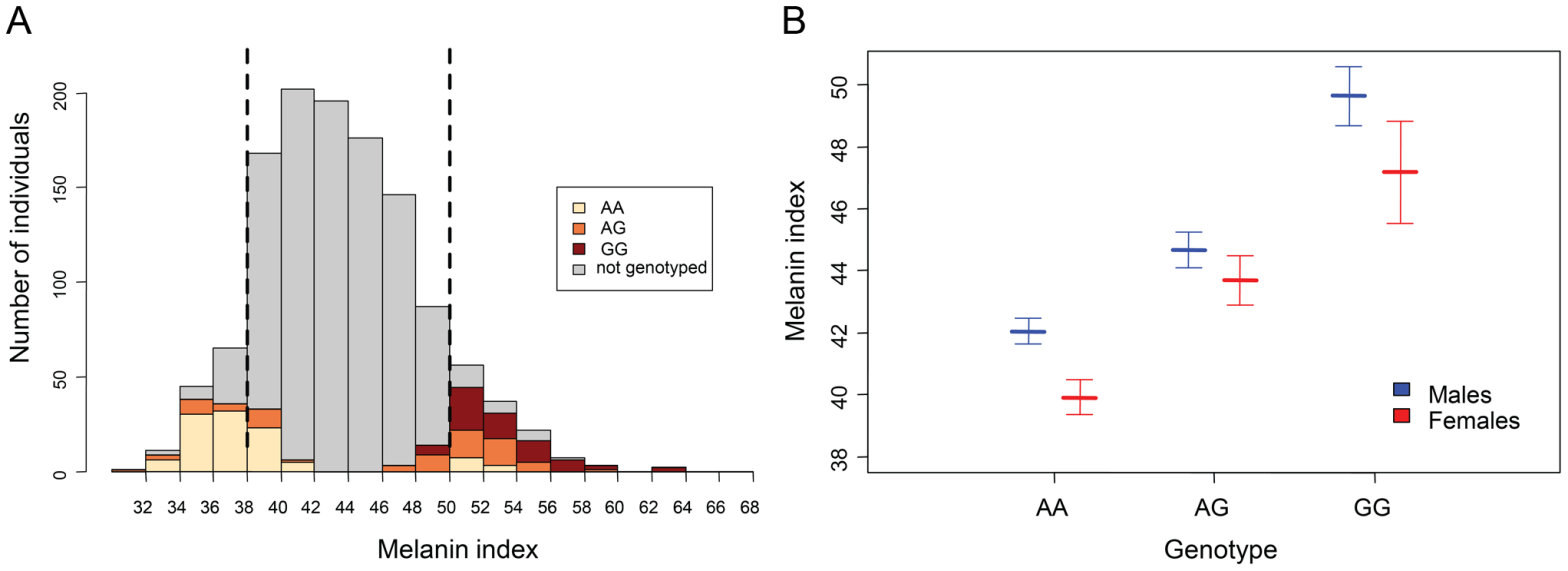
Association of rs1426654 genotypes with melanin index. (A) Distribution of melanin index (MI) in 1228 individuals of Cohort A. The two dotted black lines represent approximately 10% thresholds for the low (MI<38) and high (MI>50) MI groups, which were used to assess genotype-phenotype association using a logistic regression model. (B) Distribution of mean melanin index for the genotypes of rs1426654. The mean melanin indices for each genotype, as obtained separetely for males and females are shown together with their 95% confidence intervals, as estimated by multiple imputation model (Table S3A).

We tested the association of the rs1426654 SNP with pigmentation differences between the low (MI<38) and high (MI>50) MI groups of Cohort A (Figure 1A), using a logistic regression model. A likelihood-ratio test to discern the association of the rs1426654 SNP to skin pigmentation, in addition to the influence of sex and population (caste), showed a highly significant effect of rs1426654 genotype on skin pigmentation (p = 2.4×10^−31^) with an odds ratio of 26.2 (95% CI 12–67.5) for the A allele. Furthermore, the cross-validated Area Under the Curve (AUC) score of 0.83 suggested that this model has a high discrimination power between the low and high MI groups. In summary, most of the pigmentation differences observed between the low and high MI groups could be explained by the rs1426654 SNP.

We further aimed to estimate the effect size of the SNP. However, direct estimation of the effect size based on the samples genotyped from high or low MI group of Cohort A would only allow us to assess the effect of genotype for the extremes of pigmentation phenotype rather than for the whole distribution. Therefore, to estimate how much variation in MI could be explained by the rs1426654 SNP if all 1228 individuals in Cohort A had been genotyped, we used a multiple imputation approach based on simulations. The distribution of estimated mean MI across the genotypes, as obtained separately for males and females from the imputed dataset, is presented in Figure 1B and Table S3A. We observed that the estimated mean MI for each genotype in females was lower than that of males (Table S3A). Analysis of the imputed datasets using a General Linear Model (GLM) revealed that the effect of genotype was highly significant (p<1×10^−16^). Notably, the total variation in pigmentation (R^2^) that can be explained by the full model (including sex and genotype) was calculated to be 29% (95% CI, 24–34), while that by the SNP alone was 27% (95% CI, 22–32).

Besides the quantitative assessment of the effect size, we found that the effect of the SNP was not exactly additive. Individuals with GG genotypes were darker than expected under the additive model (Table S3B). This result is consistent with the similar mode of inheritance observed in *SLC24A5* by Lamason [4] and in other pigmentation genes, such as *KITLG* (OMIM 184745) and *SLC45A2* [7], [19].

Similar to Cohort A, our genotype-phenotype association tests on heterogeneous populations of Cohort B (Table S2), using a GLM after adjusting for sex and population, revealed that the effect of genotype was significant (p = 3.24×10^−8^). However, unlike Cohort A, where we did not observe any significant difference in mean MI of three castes (p = 0.65), the effect of population in Cohort B was highly significant (p<2.2×10^−16^).

### Geospatial distribution of rs1426654-A allele and its correlation with geography, language and ancestry component

In an attempt to map the geospatial pattern of rs1426654-A allele frequencies across South Asia, we genotyped 1054 individuals across 43 ethnic groups including major language groups and geographic regions (see Materials and Methods, Cohort C) from the Indian subcontinent. In summary, 1573 individuals from 54 distinct tribal and caste populations from all the three cohorts (A, B and C) were assessed for this polymorphism (Table S4; Figure S1). We found that the rs1426654-A allele is widely present throughout the subcontinent, although its frequency varies substantially among populations (0.03 to 1) with an average frequency of 0.53±0.32 (Table S4). To explain how the various genetic and non-genetic factors affect the geospatial distribution of the rs1426654-A allele in the Indian subcontinent, we assessed the correlation of rs1426654-A allele frequency with major geographical divisions, language families and the ancestry component detected in previous studies of Indian populations [24], [25]. However, to avoid bias due to low sample sizes in some of the populations, only data from 1446 individuals representing 40 populations were used (Table S5).

**Figure 2:**
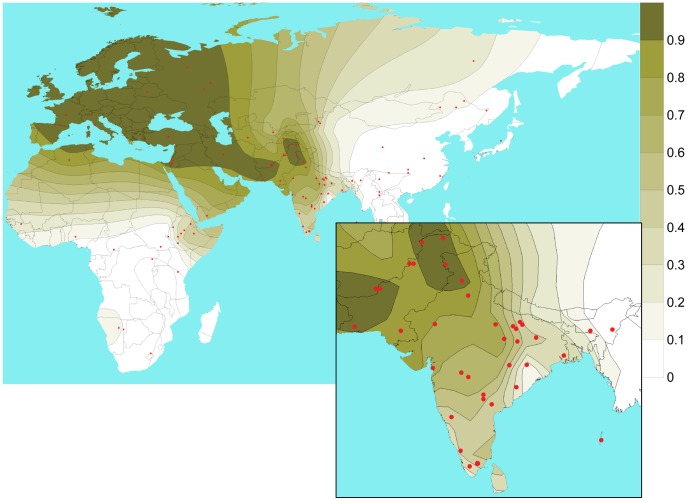
Isofrequency map illustrating the geospatial distribution of SNP rs1426654-A allele across the world. The map has been drawn based on rs1426654-A allele frequencies of 2763 subjects obtained from published datasets (Table S7) and 1446 individuals from the present study (Table S5). Red dots correspond to the sampling locations.

Although we observe a considerable local heterogeneity, there is a general trend of rs1426654-A allele frequency being higher in the Northern (0.70±0.18) and Northwestern regions (0.87±0.13), moderate in the Southern (0.55±0.22), and very low or virtually absent in Northeastern populations of the Indian subcontinent (Figure 2, Table S6). Notably, the Onge and the Great Andamanese populations of Andaman Islands also showed absence of the derived-A allele. Given the fact that one can observe a pronounced latitudinal cline for skin pigmentation across world populations, we also sought to test the observed derived-A allele frequencies in terms of absolute latitude and longitude in South Asia. We found that the rs1426654-A allele frequency in South Asia does not significantly correlate with latitude (r = 0.23, p = 0.15). However, a significant negative correlation with longitude (r = −0.49; p = 0.002) was observed.

We found that the Tibeto-Burman and the Austroasiatic language families have the lowest frequencies of the A allele ( and Table S6). The rs1426654-A allele frequency was significantly higher in Indo-European speakers than in other language groups (Table S6). In particular, there was a significant difference (p<0.001) between the A allele frequencies of the Indo-European and the Dravidian speaking groups. We found that both language and geography have a significant influence on rs1426654-A allele frequency, as revealed by Mantel tests (p<0.001).

We also studied the geospatial pattern of rs1426654-A allele frequencies at the global level using 2763 subjects from previously published data (Table S7) and 1446 individuals from the present study (Table S5). The isofrequency map illustrates high frequencies of the rs1426654-A allele in Europe, Middle East, Pakistan, moderate to high frequencies in Northwest and Central Asia, while being almost absent in East Asians and Africans with notable exceptions in Bantu (Southwest), San, Mandeka, and Ethiopians (Table S7, Figure 2). As rs1426654-A allele frequency was found to be higher in West Eurasian populations that are known to share one of the genome-wide ancestry components of South Asia [24], [25], we sought to test the correlation between the derived-A allele frequency and the proportion of the West Eurasian ancestry component (as depicted by the “light green component” in [24]) for the studied populations. For this, we used the genome-wide information available on Indian populations from literature [24]–[28] (Table S8) and relevant global reference populations to perform the ADMIXTURE run. Population structure as inferred by ADMIXTURE analysis at K = 7 is shown in Figure S2A. The proportions of k5 light green ancestry component obtained at K = 7 for the populations studied were plotted against the rs1426654-A allele frequency available for all populations and South Asia in particular (Figure S2B). As shown in Figure S2B, we obtained a significant positive correlation for South Asian populations (r = 0.90, p<0.0001) but a weak, although significant correlation when all populations sharing the k5 component (r = 0.64, p = 0.04) were considered.

### Fine-scale genetic variation of *SLC24A5*

**Figure 3:**
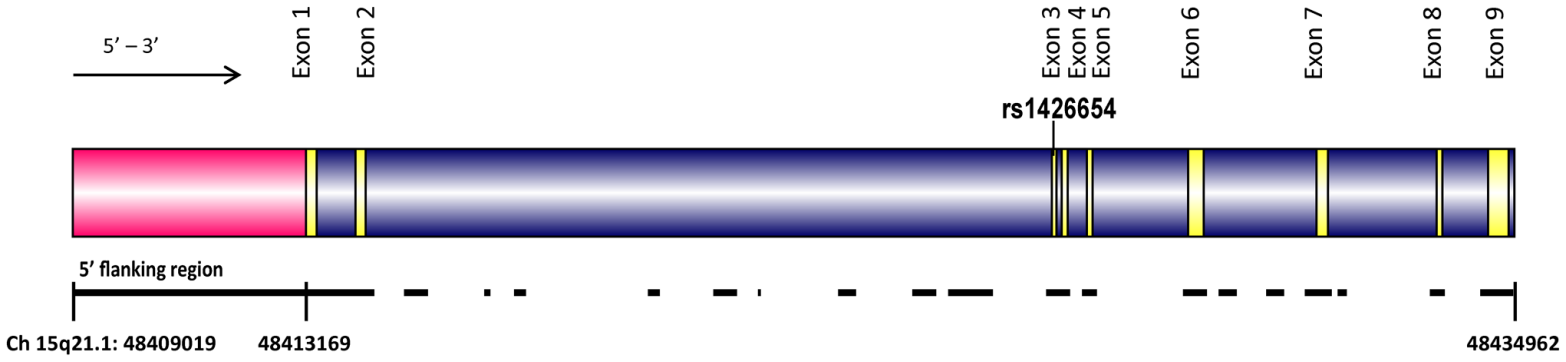
The structure of the human *SLC24A5* gene (Chromosome 15q21.1, 48409019 to 48434692). Exons of the gene are shown in yellow, introns in blue and 5′ flanking region in pink. The black lines underneath the gene show the regions resequenced in this study (total of 11741 bp) spanning 25674 bp. rs1426654 is the functional SNP located in the third exon.

We resequenced 11.74 kb of *SLC24A5* (Figure 3), covering all the nine exons (1617 bp), introns (5797 bp), 5′ flanking (4150 bp), and 3′ flanking (177 bp) regions (Figure 3) in a global sample set of 95 individuals (see Materials and Methods) grouped into 8 broad geographic regions. A total of 60 variable sites (including 23 singletons), one insertion, and one tetranucleotide repeat were identified with derived allele frequencies ranging from 0.005 to 0.39. Results of the resequencing study for these variable sites are presented in Table S9. According to dbSNP (http://www.ncbi.nlm.nih.gov/projects/SNP/) build 137 (June 2012), 21 of these 62 identified variants were novel. The insertion present in the 5′ flanking region (position 48411803) was confined to two San individuals (San 15 and San 17). Comparison of polymorphic sites across different regions revealed that the exons of *SLC24A5* are highly conserved in humans. We detected only two variable positions within exons, with rs1426654 being the only non-synonymous SNP. The other variant, a synonymous (Ser-Ser) mutation identified at exon 7 at position 48431227, was shared by four Africans. In contrast to low variation in the exonic region, a highly polymorphic tetranucleotide repeat (GAAA) was observed in the 5′ flanking region (GAAA-GA-GAAA-GAAAAA-**(GAAA)_n_**-GAAAAA-GAAAA) at position 48412029. These repeats varied from 3 to 12 copies. A detailed analysis of the repeats did not reveal any correlation with the geographical origin of the samples or the haplogroups studied, in general (Table S10). However, chromosomes belonging to haplogroup H (Figure S3), defined by the rs1426654-A allele, were associated with larger repeat lengths (7–13), albeit this association was not restricted only to them (Table S10).

**Figure 4:**
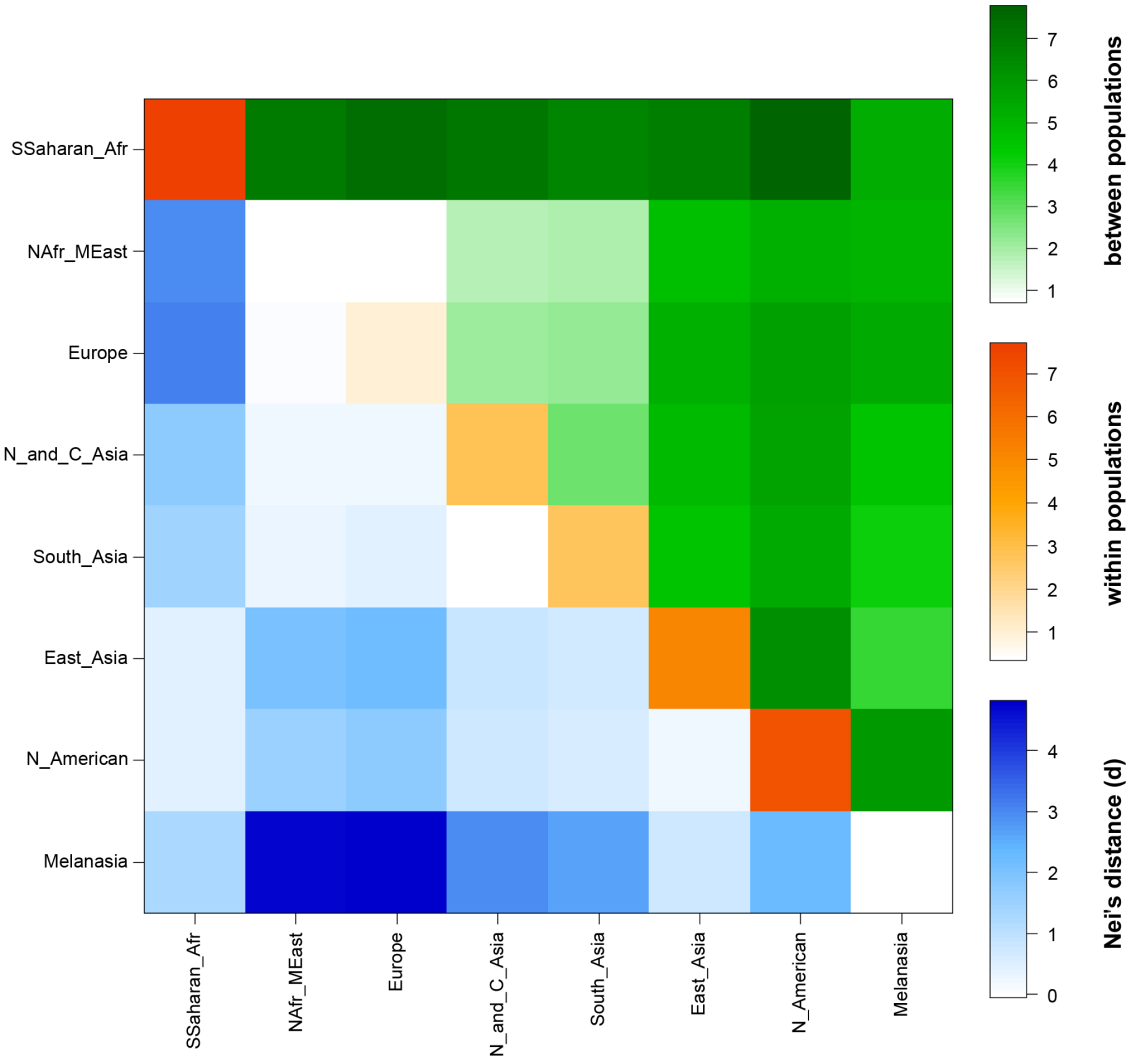
Heat map showing the intra- and inter-population variation measured by average pairwise sequence differences of the *SLC24A5* gene. The upper triangle of the matrix (green) shows average pairwise differences between populations (PiXY). The average number of pairwise differences (PiX) within each population is shown along the diagonal (orange). The lower triangle of the matrix (blue) shows differences between populations based on Nei's distance, i.e., corrected average pairwise differences (PiXY−(PiX+PiY)/2).

The nucleotide diversity estimated for the consensus resequenced region (11741 bp) was observed to be 0.00042±0.00004 (with Jukes-Cantor correction), which is low compared to the average of 0.00071±0.00042 for 647 genes resequenced in the NIEHS SNP database (http://egp.gs.washington.edu/). A sliding window approach based on similar measures (window size = 100 bp, step size = 25 bp) for the 5′ flanking region (4150 bp) sequenced revealed that the 2726–2875 region demonstrates the highest nucleotide diversity of 0.00651 (Figure S4). Various molecular diversity indices studied for the eight geographical groups are presented in Table S11 and Figure S5. Average pairwise differences observed among and within 8 different geographical regions using 11741 bp sequence data are summarized in Figure 4. Populations from regions previously reported to exhibit a high frequency of the rs1426654-A allele (North Africa and Middle East, Central Asia, South Asia and Europe; see Figure 2) show low levels of intra- and inter-population diversity in the resequenced region (Figure 4, Table S11).

### Evidence for positive selection

We tested if our sequence data supports the well-documented evidence of positive selection for *SLC24A5* in previous studies [4], [13], [20], [21], [29], [30] and whether it provides any additional evidence of selection. None of the populations tested showed significant departure from neutrality, except for Europeans, who had negative Tajima's D (p = 0.02) and Fu and Li's F* (p = 0.04) as estimated from calibrated population genetic models using COSI (Table S12).

Hence, these observations confirm that *SLC24A5* has been under strong selective pressure in Europeans. In addition to this, we also performed haplotype-based selection tests based on genome-wide data (see Materials and Methods) of 1035 individuals including 145 Indians. XP-EHH scores demonstrated that *SLC24A5* ranks among the top 10 candidate genes for positive selection in Europe, Middle East and Pakistan, and among the top 1% in Central Asia, Iran and North India (Table S13). Likewise, scores from our iHS analysis had significant empirical p-values for Central Asia and North India (Table S13). It is interesting to note that both of our haplotype-based selection tests demonstrated evidence of positive selection in North Indians, but no such evidence of positive selection was found in South Indians (Table S13). The difference in detecting selection signals from genotype and sequence data has also been pinpointed in a previous study [31].

### Phylogenetic analysis and coalescence age estimates

Firstly, a phylogenetic tree was drawn on the basis of common variants observed in our worldwide resequencing data (11.74 kb) of 95 individuals. The schematic tree representing the 8 most common haplogroups is shown in Figure S3. Haplogroup G was the most common and geographically widely spread clade, being found in 7 of the 8 geographical groups examined. Haplogroup C was confined to sub-Saharan Africans only, while the rest of the observed haplogroups were shared between African and non-African populations. We conclude that all of the 73 phased chromosomes (from Europe, sub-Saharan Africa, Middle East, South Asia, North and Central Asia) with the rs1426654-A allele form a monophyletic group because they share the same haplotype background regardless of their geographic origin. In other words, all carriers of the mutation in our global sample share it by descent. The presence of the derived A allele in sub-Saharan Africa, although in low frequencies (2/73 - one heterozygous Mandeka and one heterozygous San individual) (Figure S3) is consistent with earlier findings [32].

We estimated the coalescence time of the rs1426654 mutation at 28,100 years (95% CI - 4,900 to 58,400 years) using BEAST. Using the same mutation rate, the coalescent age estimated by rho statistics was 21,702 years ±10,282 years. Despite the different assumptions used in the two coalescent age estimation methods, both the age estimates show substantial overlap.

## Discussion

### Effect size of the rs1426654 SNP and its association with pigmentation variation in South Asia

A number of previous studies have focused on admixed populations in the search for genes that determine skin pigmentation variation in humans [4], [5], [33]–[35]. Our formal tests for association, using a large homogenous population from South India (Cohort A) as well as a heterogeneous pool of samples across India (Cohort B), demonstrated a highly significant effect of *SLC24A5* on skin pigmentation. Further analysis of Cohort A revealed that this SNP determines most of the variation between the pigmentation extremes and contributes about 22–32% of the total skin color variation, thus suggesting that *SLC24A5* plays a key role in the pigmentation diversity observed among South Asians.

Furthermore, confounding effect of population structure on the genetics of skin pigmentation, evident in Cohort B suggests that the marked population substructure of South Asians must be taken into account when genetic association studies are conducted in these populations.

### The spread of rs1426654-A allele in South Asia

Our extensive survey of rs1426654-A allele frequency in the Indian subcontinent reveals an average frequency of 0.53 with a substantial variation among populations, ranging from 0.03 to 1 (Table S4). This finding stands in contrast to the previous understanding of the spread of this allele, where a study [23] based on a cohort of 15 Indian ethnic groups sampled in the US (n = 576), estimated the average A allele frequency at 0.86, with a relatively low level of variation among populations (observed range 0.70 to 1). The most plausible cause of this discordance might be that fewer populations were included in the former study and the groups were defined by their generic linguistic affiliation in major branches of the Dravidian and Indo-European languages, rather than by finer resolution of the endogamous units. Notably, in the subset of 8 populations that could be characterized on a similar basis in both studies, the estimates of A allele frequencies did not diverge significantly in their combined averages (Table S14). Therefore, these comparisons suggest that sampling strategies are pivotal in determining the extent of genetic diversity observed in Indian populations and that sampling of expatriates may have a homogenizing effect. Moreover, the expatriates are known to represent mainly urban populations of India, which constitute only 30% (Census 2011; http://censusindia.gov.in/) of the total population of the subcontinent, and therefore are unlikely to be representative of the wealth of genetic variation harbored within the subcontinent.

### Factors shaping the complex pattern of the rs1426654-A allele in South Asia

Our quest to determine whether and to what extent the distribution of the rs1426654 derived- A allele frequency in South Asian populations correlates with language and/or geography revealed that both of these variables have a significant predictive value on allele frequencies. In particular, we found that although frequencies among populations studied vary considerably, this polymorphism has an evident geographic structure with higher frequencies of the derived allele in North and Northwest regions and a declining pattern as one moves further South and East (Table S5, Figure 2). However, when we plotted the rs1426654-A allele frequency against the geographical coordinates of our sampled populations, we found a significant correlation with longitude but not with latitude. The lack of a clear latitudinal (North-South) cline in the A allele frequency, which would have been expected under the model of natural selection, could be partly explained by the complexity of the South Asian genetic landscape, influenced by differences in population histories shaped by various micro-level migrations within the subcontinent, strict endogamy and social barriers. For example, Saurashtrians, who migrated from “Saurashtra” region of Gujarat to South India (Madurai) for work, have a relatively high rs1426654-A allele frequency of 0.70. It is believed that those Saurashtrians presently dwelling in Madurai were invited by Nayak kings for their expertise in silk-weaving [36]. Similarly, Toda have higher A allele frequency (0.86) compared to Kurumba (0.20), their geographical neighbors, most likely due to their higher proportion of West Eurasian ancestry which is supported by Y chromosome evidence [37]. Notably, Brahmins, irrespective of their geographic source (North, Central or South India) have higher A allele frequency (Table S5). Conversely, the higher longitudinal correlation could be due to the fact that Tibeto-Burman and Austroasiatic speakers are characterized by very low A allele frequency (Table S6) because of their East Asian ancestry [26], [38]. Therefore, their inclusion in our sampling might have resulted in the inflation of the longitudinal correlation coefficient.

### Coalescence age estimate of the rs1426654-A allele

Although the last decade has witnessed significant improvement in the understanding of the genetic basis of skin pigmentation, our knowledge about the exact mechanisms behind the evolution of light skin in humans is still incomplete. The genetic evidence that has accumulated till date suggests a complex evolutionary history for skin pigmentation. It has been argued that natural selection in response to UVR had a causative role in the evolution of light skin color at high latitudes [8], [39], [40]. Evidence of population-specific signatures of selection of pigmentation genes at different timescales suggests that the evolution of light skin was not a one-step process [41], [42] but a consequence of multiple events or episodes during human evolution. It appears that some of the mutations which have been associated with light skin started to accumulate relatively early in modern human history in the proto-Eurasian populations following the Out-of-Africa expansion, whereas other mutations arose after the divergence of East and West Eurasian populations [4], [19], [29], [41].

Hence, studies focusing on the time-scale of genetic changes in pigmentation genes are vital for understanding the complex evolutionary history of human skin pigmentation. Therefore, in this study, we focused on providing an age estimate of the rs1426654 mutation, which has a major effect on skin pigmentation in West Eurasian and South Asian populations. Notably, previous studies providing age estimates for this locus have been mostly confined to the estimation of onset of selective sweep rather than the coalescence time of the mutation. A study of extended haplotype homozygosity in HapMap populations estimated that the most intense signals of selection detected in European and East Asian populations are found in haplotypes which extend 0.52 cM on average in length [20]. Assuming a star-shaped genealogy and a generation time of 25 years, the authors dated the peak of these signals to ∼6.6 KYA [20]. They also observed that the second-longest haplotype (1.15 cM) in Europe includes *SLC24A5*, where rs1426654-A was found to be fixed. Using the same formula used by Voight [20] to date the average peaks of selection signals in Europe and East Asia, the selective sweep specifically at *SLC24A5* in the HapMap European sample can be dated to ∼3 KYA. Besides this, a recent study by Beleza [42], focusing on analyses of diversity in microsatellite loci, estimated that the selective sweep at *SLC24A5* occurred around 11.3 KYA (95% CI, 1–55.8 KYA) and 18.7 KYA (5.8–38.3 KYA) under additive and dominant models, respectively [42].

Our Bayesian coalescent age estimate of the rs1426654-A allele at ∼28 KYA (95% HPD, 5–58 KYA), as well as the rho-based estimate at 21.7 (±10.3) KYA, are older in their point estimates than both of the above selective sweep date estimates, although these age estimates have broad and overlapping error margins. This finding is not surprising because sweeps can also operate on standing variation. Besides this, both our rho-based point estimate and Bayesian mean age estimate postdate the estimated time of the split between Europeans and Asians calculated by Scally [43] using a similar mutation rate. Although our confidence intervals cannot rule out entirely the possibility of older dates (>28 KYA), our findings are broadly consistent with the evolutionary model of skin pigmentation proposed in earlier studies [41], [42], [44]. It appears that the most plausible scenario is that light skin evolved as an adaptation to local environmental conditions as humans started moving to northerly latitudes, with the initial phase of skin lightening occurring in proto Eurasian populations, while genetic variation in *SLC24A5* formed the later phase which led to lighter skin in Europeans and South Asians, but not East Asians. This was followed by a European-specific selective sweep, which favored the rapid spread of this mutation in these populations. Our coalescence age estimates of 28 KYA (95% HPD 5–58 KYA) show wide margins, also evident in the earlier sweep date estimates for the gene [42]. This can be due to the fact that the power of our analysis was limited by the need to reduce our sequence range to a subset of sites from a region with sufficiently high LD around the rs1426654-A allele and very low level of sequence variation. Therefore, we speculate that narrowing down the coalescence age estimates and specifying the geographic source of the rs1426654-A allele will depend rather on the success of ancient DNA studies than on more extensive sequencing.

### Evidence for positive selection

Earlier studies have highlighted *SLC24A5* as one of the top candidate genes demonstrating evidence for positive selection in Europeans [4], [13], [20], [21], [29], [30] and in Middle Eastern and Pakistani populations from South Asia [13], [29] on the basis of either F_ST_ or extended haplotype homozygosity from genotype data. Here, relying on our previous scans of extended haplotype homozygosity on Indian populations [24], we note that both XP-EHH and iHS suggest that positive selection has occurred in North Indian (within top 5% and top 1% respectively) but not in South Indian populations. One possible explanation for the regional differences in empirical ranks of the *SLC24A5* in India could be the “melanin threshold” hypothesis [45]. According to this hypothesis, natural selection affects the variation in pigmentation phenotype only up to a certain adaptive optimum, beyond which individuals may show variation that is subject to other factors such as admixture, genetic drift etc. However, differently from the expectations of this hypothesis, we do observe high range of melanin indices both in North and South Indian populations of Cohort B (Table S2). Furthermore, the high positive correlation of rs1426654-A allele with the light-green South Asian ancestry component (Figure S2A) advocates that the rs1422654-A allele frequency patterns in India could be also explained by demographic history of the populations in addition to selection. It is also possible that while XP-EHH and iHS tests have increased power to detect selection signatures associated with high allele frequencies, the low ranking position of *SLC24A5* in selection scans of South Indians is due to the overall lower frequency of the rs1422654-A allele.

Therefore, the complex patterning of light skin allele in India and its correlation with geography, language, and ancestry component observed in the present study, portrays an interesting interplay between selection and demographic history of the populations. This stands in contrast to Europe where the frequency of the light skin associated allele of *SLC24A5* has almost reached to fixation and seems to be attributable solely to natural selection. This aspect of skin pigmentation variation observed in South Asians is pivotal in understanding the different mechanisms that contribute to the global skin pigmentation variation and in further understanding of this complex phenotypic trait.

To summarize, we have provided evidence using a homogeneous cohort that the rs1426654 SNP plays a key role in skin pigmentation variation in South Asia. We have shown that the rs1426654-A allele is widespread in the Indian subcontinent and its complex pattern is a result of combination of processes involving selection and demographic history of populations, influenced by their linguistic and geographic affiliations. Phylogenetic analyses of resequencing data confirm that the rs1426654-A allele in West Eurasian and South Asian populations occurs on the same haplotype background. Both sequence and genome-wide genotype data confirm evidence of positive selection in Europeans, while the latter supports further evidence of selection in populations of Middle East, Pakistan, Central Asia and North India but not in South India. We date the coalescence of the light skin allele (rs1426654-A) to 22–28 KYA (95% CI, 5–58 KYA). However, since this allele has become fixed in many populations across its current distribution, we propose that ancient DNA research might have greater potential to improve our understanding of when and where it first appeared.

## Materials and Methods

### Ethics statement

This study was approved by the Research Ethics Committee of the Estonian Biocentre, Tartu, Estonia and the Institutional Ethical Committee (IEC) of the Centre for Cellular and Molecular Biology, Hyderabad, India. All recruited individuals were >18 years of age and their ethnic origin was determined via personal interviews. Written informed consent was obtained from all participants.

### Subjects

Skin pigmentation was measured using DermaSpectrometer (Cortex Technology, Hadsund, Denmark). Erythema (E) and melanin index (MI) readings were taken from the upper inner arm (medial aspect) [46]. For a subset of individuals, additional measurements were taken from the forehead, representing the most tanned or sun-exposed region of the skin. However, only MI readings from the upper inner arm were used for association analyses. DNA was isolated either from blood or saliva (using Oragene DNA kits, Canada). The study involved three distinct cohorts, A, B and C. Sampling locations of these cohorts are shown in Figure S1.

Cohort A included 1228 randomly recruited individuals from three major agricultural castes (Kapu, Naidu and Reddy) of Andhra Pradesh, India. For all the above individuals, MI readings were taken from the right and left upper inner arm and their mean was calculated to determine each individual's MI. Following the phenotypic screening, thresholds were set for the “low” (MI<38) and “high” (MI>50) MI groups respectively, representing approximately the top and bottom 10% of the MI distribution, for collection of DNA samples (Figure 1A). Eighty-four out of 120 individuals from the low MI group and 102 out of 127 individuals from the high MI group were genotyped successfully. The 10% threshold was implemented after an initial pilot study, following which the values were continuously redefined as the sample collection progressed. Consequently, during the fieldwork, DNA from 56 individuals was collected outside the determined thresholds (MI 38–50). Therefore, in summary, 242 individuals (189 males, 53 females) from this cohort were genotyped for the rs1426654 SNP (Table S1).

Cohort B comprised of 446 individuals, including 10 caste and tribal populations of Tamil Nadu, Maharashtra and Haryana states of India. For each individual, three readings of MI were taken from the right upper inner arm and the values were averaged. Out of these, 277 individuals (246 males and 31 females) were genotyped (Table S2).

Cohort C included 1054 individuals, representing 43 endogamous populations from different ethnic backgrounds, language families (Dravidian, Indo-European, Austroasiatic, Tibeto-Burman speakers), castes, tribes, with their geographical locations covering most of the states. No records for MI were available for this cohort.

In summary, 1573 individuals from 54 distinct tribal and caste populations including all the three cohorts (A, B and C) were assessed for the rs1426654 polymorphism (Table S4 and Figure S1). A detailed description of the geographic location, linguistic affiliation and socio-cultural background of each cohort is given in Tables S1, S2 and S4S4. Populations from Cohort A and Cohort B with MI readings were used for genotype-phenotype analyses and genotyping results from all three cohorts (A, B and C) were used to map the spread of rs1426654-A allele and test its correlation with language, geography and ancestry component.

For the resequencing study, we designed a global panel comprising of 95 individuals. This included 70 subjects from HGDP-Centre d'Étude du Polymorphisme Humain (HGDP-CEPH) worldwide panel [47], and additionally 3 Europeans, 18 Indians, and 4 Central Asians to cover the underrepresented regions of the CEPH panel. For population-level analyses, these 95 individuals were broadly classified into 8 major groups based on their geography and ethnicity: sub-Saharan Africa (n = 22), North Africa/Middle East (n = 7), Europe (n = 11), North and Central Asia (n = 7), South Asia (n = 23), East Asia (n = 14), Native Americans (n = 4) and Melanesia (n = 7). List of the populations included in the resequencing project, representing these regions is given in Table S9.

### Genotyping of the rs1426654 SNP

A 443 bp region of *SLC24A5* flanking the rs1426654 SNP was amplified by PCR using s.E3,4F and s.E3,4R primers (Table S15). The cycling protocol consisted of 96°C for 3 min, 32 cycles of 96°C for 30 s, 57°C for 30 s, 72°C for 1 min and final extension at 72°C for 5 min. The PCR product was then either directly sequenced or digested overnight at 37°C using *Hin*6I restriction endonuclease enzyme. All digested products were run on a 3% agarose gel. The products for sequencing were run on 3730XL DNA Analyzer (Applied Biosystems, Foster City, CA) using Big Dye Terminator sequencing kit (v3.1 Applied Biosystems).

### Genotype-phenotype association analyses

The effect of the functional *SLC24A5* SNP (rs1426654) on skin pigmentation differences between low (<38 MI) and high (>50 MI) MI groups of Cohort A was tested using a logistic regression model. For this, we compared a model that included sex and population (caste) as predictors to a model in which the genotype was added as an independent variable. An association between SNP and melanin index was tested using a likelihood-ratio test after adjusting for sex and population and, assuming additivity, odds ratio was calculated for the rs1426654-A allele. Furthermore, we calculated the cross-validated Area Under the Curve (AUC) value to quantify how accurately this polymorphism predicts the occurrence of an individual in the low or high MI group, using the R package caret [48].

To estimate the effect size of the SNP, we used a simulation-based approach known as multiple imputation [49]. This method uses regression models and Bayesian sampling to impute missing values conditional on other predictors. Using random imputations, 1000 complete datasets were generated. The desired analysis was performed on each dataset using methods based on complete data. Results were pooled to derive corrected point estimates and inference [49], [50]. Using this methodology, we estimated the mean MI for each genotype separately for males and females. We also estimated the coefficient of determination (R^2^) for the full model which included sex and genotype, and the variation of melanin index that can be explained by rs1426654 SNP alone. We tested the effect of genotypes on melanin index using a generalized linear model (GLM). All the above stated analyses were performed using the R package MICE 2.9 [50].

For randomly collected samples (Cohort B), similarly, the effect of rs1426654 genotypes on melanin index was assessed using a GLM. Furthermore, the effect of the genotype in the cohort studied was tested using an additive model. All statistical analyses were performed using the R computing package (version 2.15.2.1) (http://www.r-project.org/).

### Geospatial distribution of the rs1426654-A allele

To visualize the geospatial pattern of the rs1426654 SNP in South Asia and to compare it with other populations across the world, an isofrequency map was generated using 1446 individuals genotyped across all three cohorts (Table S5) and 2763 subjects from previously published datasets (Table S7). The isofrequency map was drawn using Surfer 8.0 (Golden Software Inc, Golden, Colorado).

To test the distribution of the rs1426654-A allele across different language families and geographical coordinates, all of the individuals genotyped under the three cohorts were grouped into 7 geographical zones and 4 major language families pertinent to India (Table S6). Some populations were regrouped with their geographical neighbors of same ethnicity (Table S5). Populations that could not be grouped and had low sample size (n<15) were excluded. Therefore, data from 1446 individuals representing 40 populations were used for the linguistic and geographical analyses (Table S5 and S7). The rs1426654-A allele frequency was also assayed across the geographical coordinates (absolute latitude and longitude) using Pearson's correlation test. A Mantel test was used to examine the interaction of the allele frequencies with geography and language. For this, the genetic distance matrix (based on F_ST_) was generated in Arlequin 3.5.1.3 [51] and the geographical matrix was calculated from geographic coordinates. For the language matrix, we used the binary approach by coding populations speaking a language from the same language family as 0 and different language family as 1. A Mantel test was performed using Arlequin with 10,000 permutations.

### Correlation between rs1426654-A allele frequency and South Asian ancestry proportion

We tested the correlation between the derived rs1426654-A allele frequency and the proportion of the ancestry component that South Asian populations share with West Eurasians (as depicted by the “light green component” in [24]). For this, genome-wide datasets on Indian populations available from literature [24]–[28] and relevant global reference populations were combined and subjected to structure-like analysis using ADMIXTURE [52] to determine the proportions of the hypothetical ancestral populations using the methods described by Metspalu [24]. A list of the populations included in the run and their source from the literature is given in Table S8. We ran ADMIXTURE 100 times from K = 2 to K = 9 to monitor convergence between individual runs. Log-likelihood scores suggested that the global maximum was reached at K = 7. Population structure of the studied populations as inferred by ADMIXTURE analysis at K = 7 using 98,189 SNPs is shown in Figure S2A. The proportions of the k5 light green ancestry component (Figure S2A) at K = 7 were then extracted and compared with rs1426654-A allele frequency, for those world and South Asian populations for which the rs1426654 frequency was available, using Pearson's correlation test.

### Resequencing project

A total of 11.74 kb region of *SLC24A5* comprising exons (1617 bp), introns (5797 bp), 5′ flanking (4150 bp), and 3′flanking (177 bp) regions spanning over 25.6 kb (48409019–48434692) was resequenced (Figure 3) in 95 multiethnic individuals using 31 pairs of validated primers (Table S15). PCR products were purified with Exo-SAP prior to sequencing. Bidirectional sequencing for each fragment was performed using Big Dye Terminator sequencing kit (v3.1 Applied Biosystems) and run on 3730XL DNA Analyzer (Applied Biosystems, Foster City, CA). The sequences were then assembled and analyzed by Seqscape ver 2.5 (Applied Biosystems). BIOEDIT 7.1.3 was used to align the sequences to the NCBI Reference Sequence (NG_011500.1; 28421 bp). Variants were annotated with SNPs included in dbSNP build 137, June 2012. All of the variants were confirmed by manual inspection. The sequences were phased using PHASE 2.1.1 implemented in DnaSP 5.10.01 [53]. Sequence diversity measures (π and θ) were computed using DnaSP [53] and Arlequin 3.5 [51] was used to perform the interpopulation and intrapopulation analyses.

### Tests of selection

For resequenced data, we tested for the effects of selection using Tajima's D [54], Fu and Li's D* and F* [55] statistics, calculated in DnaSP 5.10.01 [53]. All the tests were performed under the standard assumption of constant population size. However, since these tests are known to be strongly influenced by population history, the significance of the results was also estimated by means of coalescent simulations using the COSI 1.2.1 software with the best-fit population model [56]. We performed 10,000 replicates. Coalescent simulations were conditioned on a specific mutation and recombination rate. We used a mutation rate of 5×10^−10^ substitutions/site/year, as reported by Scally and Durbin [43]. Estimates for the local *SLC24A5* recombination rate were obtained from HapMap Build 37 [57] and the length of simulated sequence matched that of the resequenced region (11741 bp). In the absence of an appropriate demographic model and empirical distribution, we have used the evolutionarily closest population implemented in COSI to assess the significance.

For selection analyses based on genome-wide genotype data, we used a merged data set of Illumina Infinium 650K, 610K and 660K available for 145 Indians and worldwide samples including Bantu (n = 19), Middle East (n = 133), Europe (n = 100), Central Asia (n = 77), Iran (n = 20), Pakistan (n = 165), East Asia (n = 211), Oceania (n = 27) from published datasets. Two haplotype-based selection tests, Cross-Population Extended Haplotype Homozygosity (XP-EHH) and Integrated Haplotype Scores (iHS), were used to assess the empirical rank of the *SLC24A5* in the haplotype homozygosity scans performed across the genome in each of the 8 world regions. iHS and XP-EHH statistics were calculated using code by Joseph Pickrell, available at hgdp selection browser (http://hgdp.uchicago.edu/). The analyses were based on a genome scan of 13,274 windows of size 200 kb each. Unphased SNP data were retrieved for the genomic window containing *SLC24A5* (chromosome 15:46.2–46.4 Mb (Build 36/hg18) and compared to the empirical distribution of other windows across the genome. Yoruba was used as the reference population in XP-EHH analyses. Data were phased using Beagle 3.1. [58].

### Phylogenetic analysis and coalescent age estimates

We estimated the phylogeny of *SLC24A5* haplotypes based on sequences of 11.74 kb for our diverse set of 95 individuals. For this, haplotypes were inferred from the genotype data using PHASE v.2.1.1 [59]. A neighbor-joining phylogenetic tree was constructed from these data using MEGA 5 [60]. A schematic tree representing the eight most common branches of the haplotype tree is shown in Figure S3.

We estimated the age of the rs1426654 mutation using 8837 bp of the *SLC24A5* gene. This region was determined by the largest linkage-disequilibrium block identified by the four-gamete rule algorithm, using a minimum D' value of 0.8, as implemented in Haploview 4.2 [61]. Coalescence times were estimated using Bayesian phylogenetic analysis in BEAST 1.7.0 [62]. The analysis was conducted on a dataset of 73 sequences carrying the rs1426654-A allele. We further restricted our dataset to 7837 bp comprising of third codon sites, introns and flanking regions. The F81 [63] nucleotide substitution model was selected as the best-fit model using the Bayesian information criterion in Modelgenerator [64]. The analysis was performed using a strict molecular clock and the Bayesian skyride coalescent model [65]. The molecular clock was calibrated using the mutation rate reported by Scally and Durbin [43], with a mean of 5×10^−10^ mutations/site/year and a standard deviation of 5.1×10^−11^. Posterior distributions of parameters were estimated by Markov chain Monte Carlo simulation, with samples drawn every 1000 steps over a total of 10,000,000 steps. Three independent runs were conducted to check for convergence to the stationary distribution and the first 1000 samples were discarded as burn-in. Sufficient sampling of parameters was evaluated using Tracer 1.5 [66] and samples from independent runs were combined. Sampled posterior trees were summarized to generate a maximum-clade-credibility tree. Statistical uncertainty in age estimates is reflected by the 95% credibility intervals.

We also estimated the coalescent times using the rho statistics [67] in Network 4.6 (http://www.fluxus-engineering.com/sharenet.htm) assuming a rate of 5×10^−10^ substitutions/site/year [43] and using sequence length of 8837 bp. The standard deviation was calculated according to Saillard [68].

## Supporting Information

Figure S1 Sampling locations for the present study. Map represents location of samples collected from different parts of Indian subcontinent encompassing populations of different ethnic background, language families, castes and tribes. Populations from cohorts A and B, shown in brackets, were assessed for melanin index, while the rest from Cohort C have only genotype information.
(TIF)

Figure S2 Correlation between rs1426654 A allele frequency and ancestry component. (A) Population structure inferred by ADMIXTURE analysis at K = 7. (B) Graphs showing correlation between rs1426654-A allele frequencies and light green (k5) ancestry component of the above analysis using all (North Africa/Middle East, Europe, Caucasus, Central Asia) populations in the left panel, and 27 ethnic groups from South Asia in the right panel (Hazara, Pathan, Burusho, Balochi, Brahui, Makrani, Sindhi, Gujaratis, Bhil and Meghawal, Kashmiri Pandits, Uttar Pradesh (UP) Brahmins, Kshtriya, Chamar, Dharkar, Dusadh, Kanjar, Kol, Uttar Pradesh (UP) low caste, Tharu, Gond, Naidu, Kurumba, Paniya and Malayan, Asur and Ho, Gadaba and Savara, Garo and Naga and Khasi).
(TIF)

Figure S3 Schematic tree representing the phylogenetic relationships among the samples studied in resequencing project, with haplogroup H being defined by the non-synonymous SNP rs1426654. The numbers denote the frequencies of the chromosomes in each haplogroup by the 8 geographical regions studied.
(TIF)

Figure S4 Graph showing nucleotide diversity pi (π) in the 5′ flanking region (4150 bp). Sliding window with length = 100 bp and step size = 25 bp was used to generate the graph.
(TIF)

Figure S5 Haplotype diversity indices (θ) of 8 geographical regions included in the study. The solid lines represent values of diversity indices (θ) according to Table S11. The dashed lines of the same color show standard deviations for the respective estimates.
(TIF)

Table S1 Sample description of individuals in Cohort A with rs1426654 genotyping results.
(XLSX)

Table S2 Sample description for individuals in Cohort B with rs1426654 genotyping results.
(XLSX)

Table S3 Effect of rs1426654 genotypes on skin pigmentation variation among individuals of Cohort A. (A) Estimated average melanin index (MI) for *SLC24A5* rs1426654 genotypes. (B) Difference in estimated mean melanin index (in melanin units) for the rs1426654 genotypes.
(XLSX)

Table S4 Sample description of populations under all the three cohorts (A–C) and their average rs1426654-A allele frequency.
(XLSX)

Table S5 rs1426654-A allele frequency of populations included in geographic and linguistic analyses.
(XLSX)

Table S6 Average rs1426654-A allele frequency according to their linguistic and geographic divisions.
(XLSX)

Table S7 rs1426654-A allele frequency across the world populations based on published datasets.
(XLSX)

Table S8 List of populations included in the ADMIXTURE run along with their geographic region and source of study.
(XLSX)

Table S9 Description of the variants identified in the *SLC24A5* resequencing project.
(XLSX)

Table S10 Variation among the resequencing project samples, for a tetranucleotide (GAAA) repeat at genomic position 48412029 in 5′ flanking region of the *SLC24A5* gene.
(XLSX)

Table S11 Estimates of *SLC24A5* nucleotide diversity measures among and within 8 geographic regions of the world.
(XLSX)

Table S12 Tests of neutrality for 8 geographic regions based on resequencing data.
(XLSX)

Table S13 Genome-wide rankings of the *SLC24A5* gene in haplotype homozygosity tests across world populations.
(XLSX)

Table S14 Comparison of the rs1426654 A allele frequencies of the current study with the study published by Pemberton et al. (Pemberton et al. 2008) [23].
(XLSX)

Table S15 List of primers used in the present study.
(XLSX)
