# Supplementary figures and images for: The Light Skin Allele of SLC24A5 in South Asians and Europeans Shares Identity by Descent
Source: PLoS Genet. 2013 Nov 7;9(11):e1003912. doi: 10.1371/journal.pgen.1003912 (PMC3820762; doi:10.1371/journal.pgen.1003912)

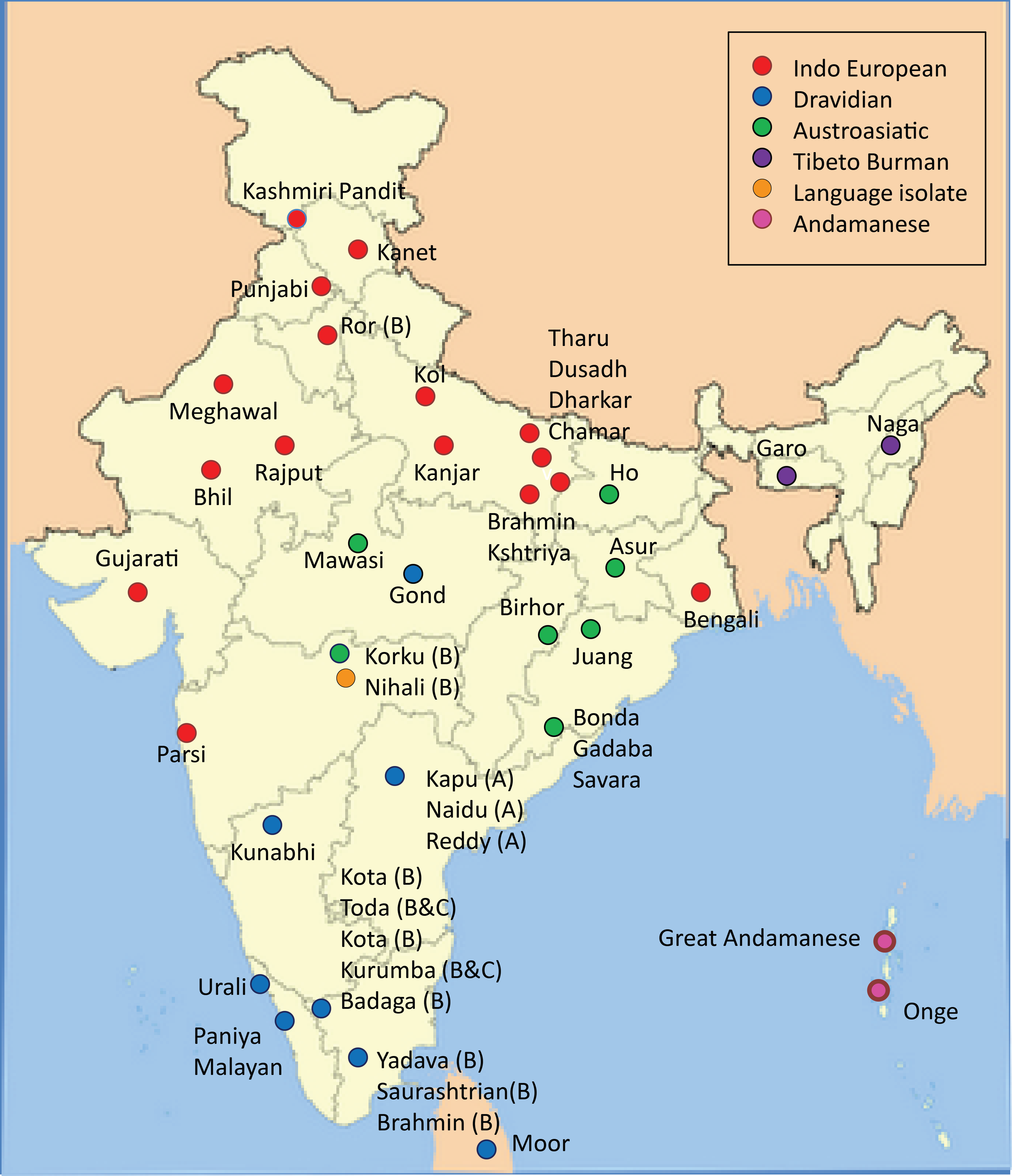

Supplement: Figure S1 — Sampling locations for the present study. Map represents location of samples collected from different parts of Indian subcontinent encompassing populations of different ethnic background, language families, castes and tribes. Populations from cohorts A and B, shown in brackets, were assessed for melanin index, while the rest from Cohort C have only genotype information. (TIF) [file pgen.1003912.s001.tif]

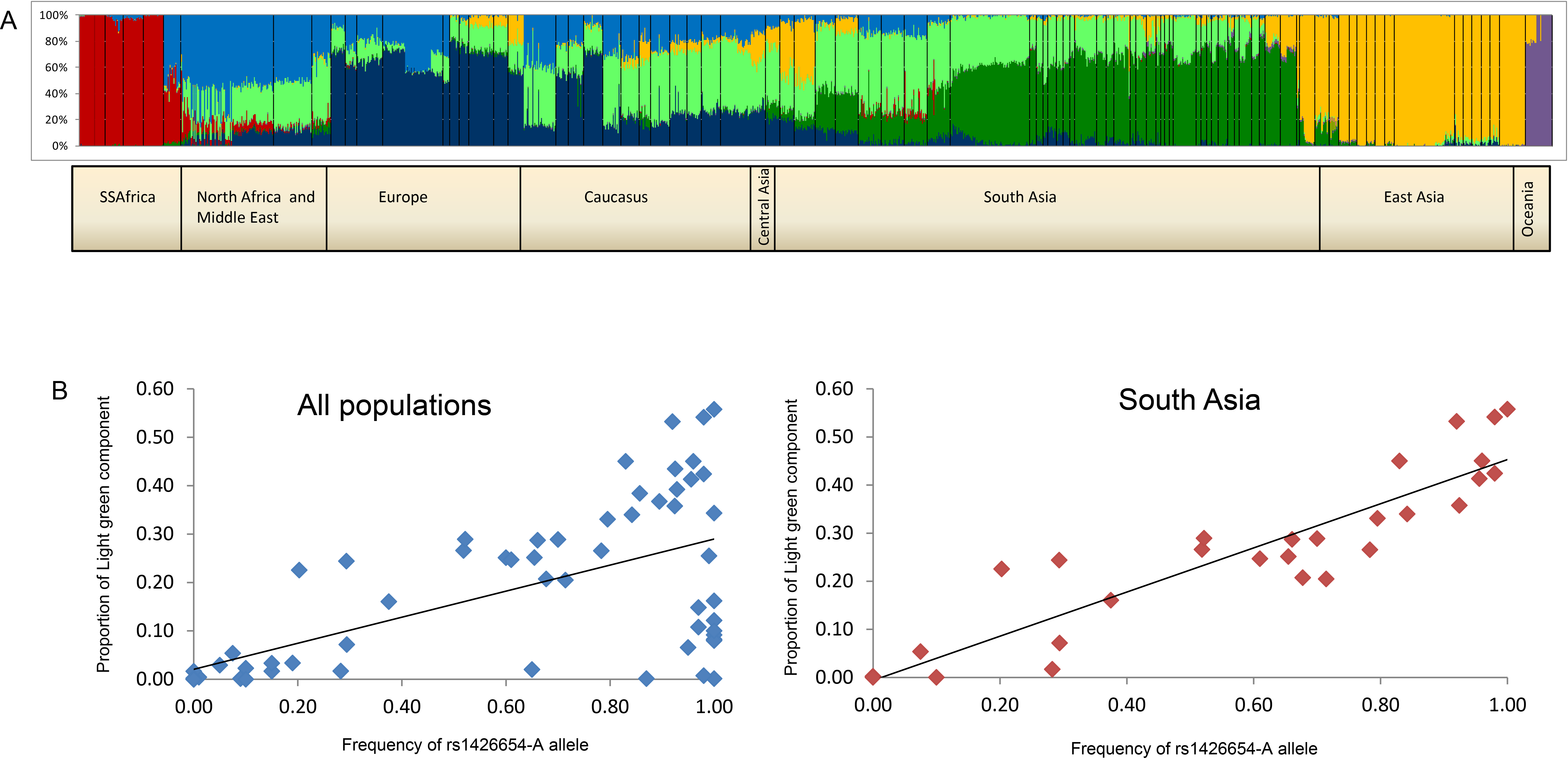

Supplement: Figure S2 — Correlation between rs1426654 A allele frequency and ancestry component. (A) Population structure inferred by ADMIXTURE analysis at K = 7. (B) Graphs showing correlation between rs1426654-A allele frequencies and light green (k5) ancestry component of the above analysis using all (North Africa/Middle East, Europe, Caucasus, Central Asia) populations in the left panel, and 27 ethnic groups from South Asia in the right panel (Hazara, Pathan, Burusho, Balochi, Brahui, Makrani, Sindhi, Gujaratis, Bhil and Meghawal, Kashmiri Pandits, Uttar Pradesh (UP) Brahmins, Kshtriya, Chamar, Dharkar, Dusadh, Kanjar, Kol, Uttar Pradesh (UP) low caste, Tharu, Gond, Naidu, Kurumba, Paniya and Malayan, Asur and Ho, Gadaba and Savara, Garo and Naga and Khasi). (TIF) [file pgen.1003912.s002.tif]

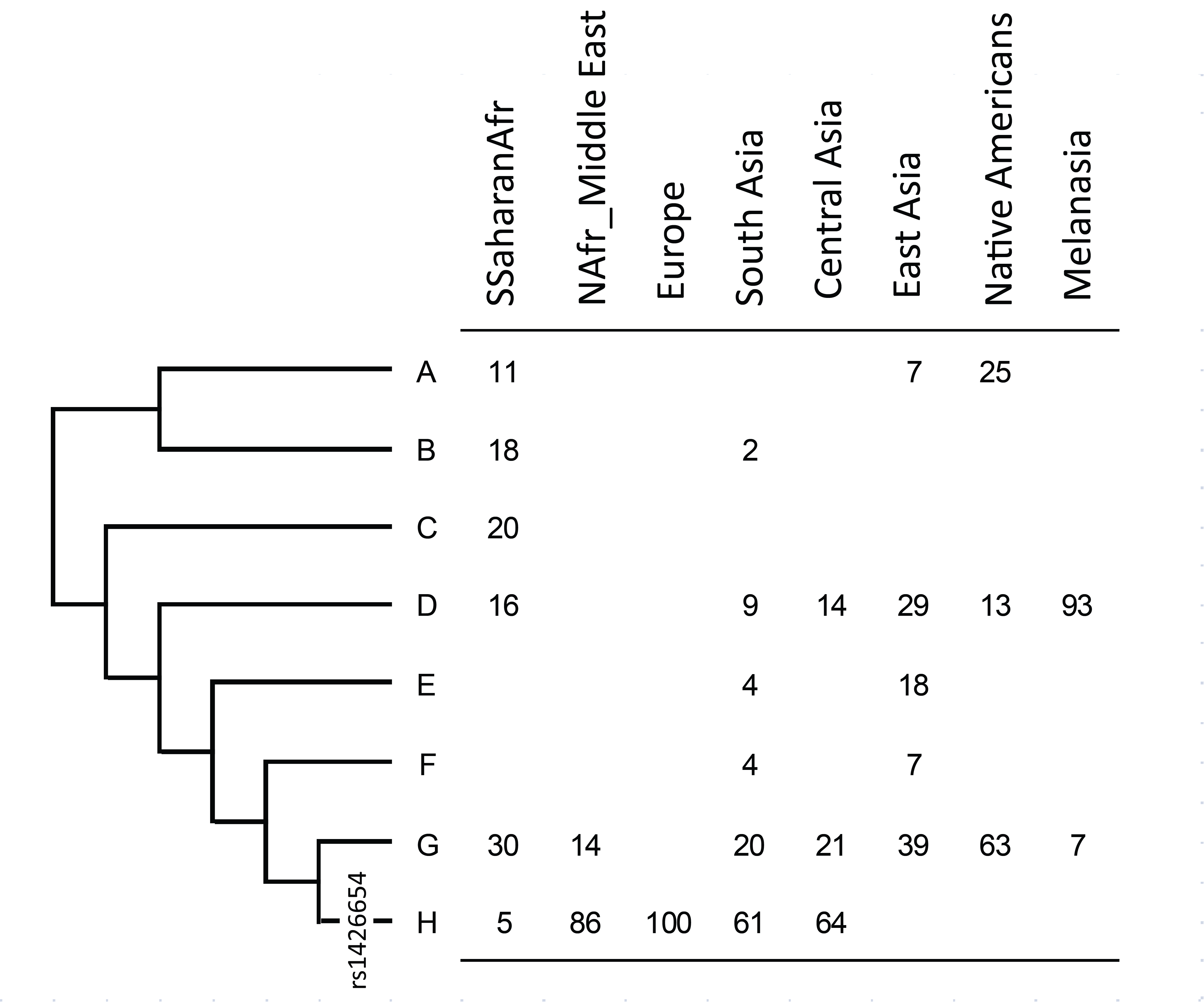

Supplement: Figure S3 — Schematic tree representing the phylogenetic relationships among the samples studied in resequencing project, with haplogroup H being defined by the non-synonymous SNP rs1426654. The numbers denote the frequencies of the chromosomes in each haplogroup by the 8 geographical regions studied. (TIF) [file pgen.1003912.s003.tif]

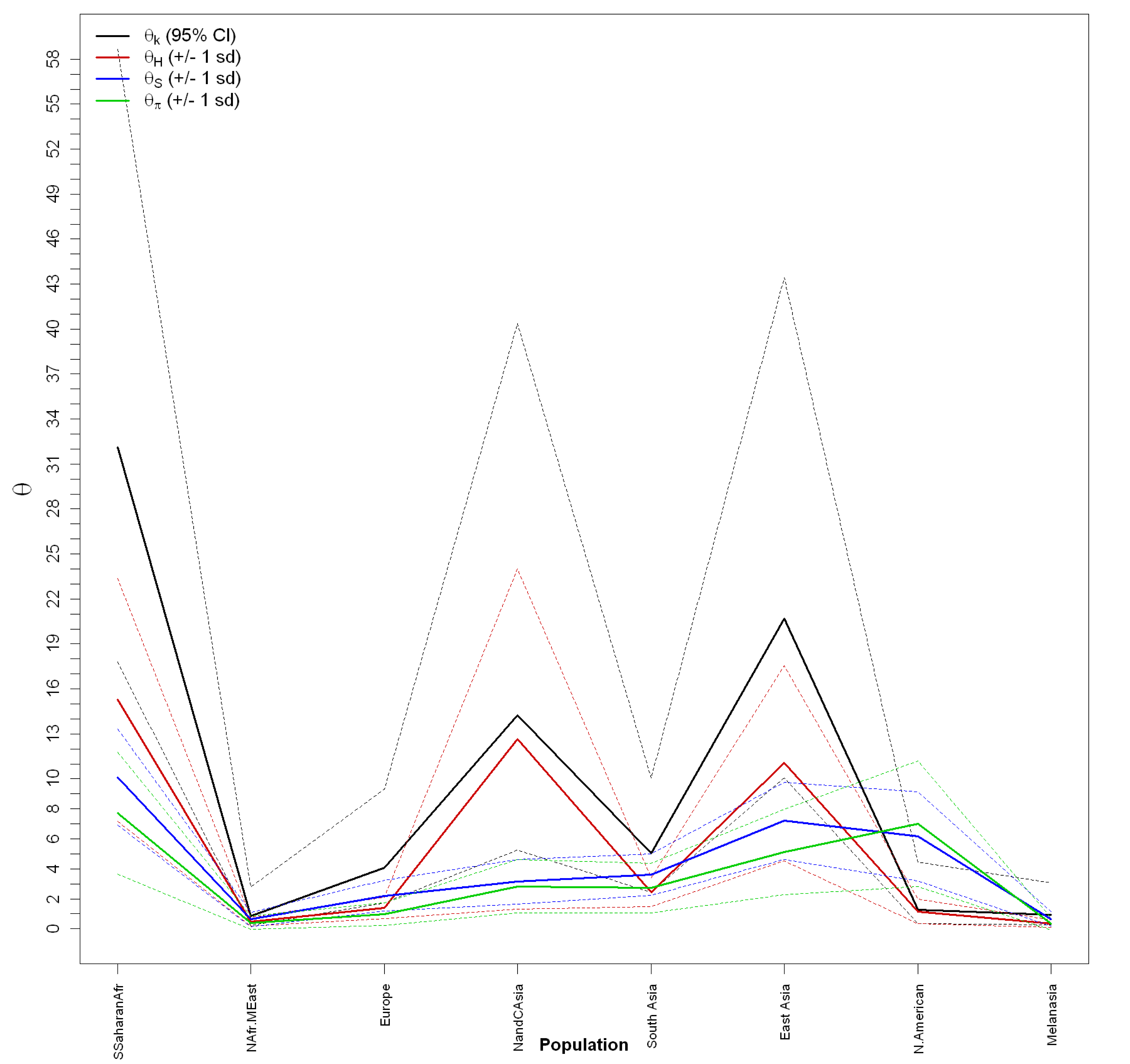

Supplement: Figure S5 — Haplotype diversity indices (θ) of 8 geographical regions included in the study. The solid lines represent values of diversity indices (θ) according to Table S11. The dashed lines of the same color show standard deviations for the respective estimates. (TIF) [file pgen.1003912.s005.tif]
